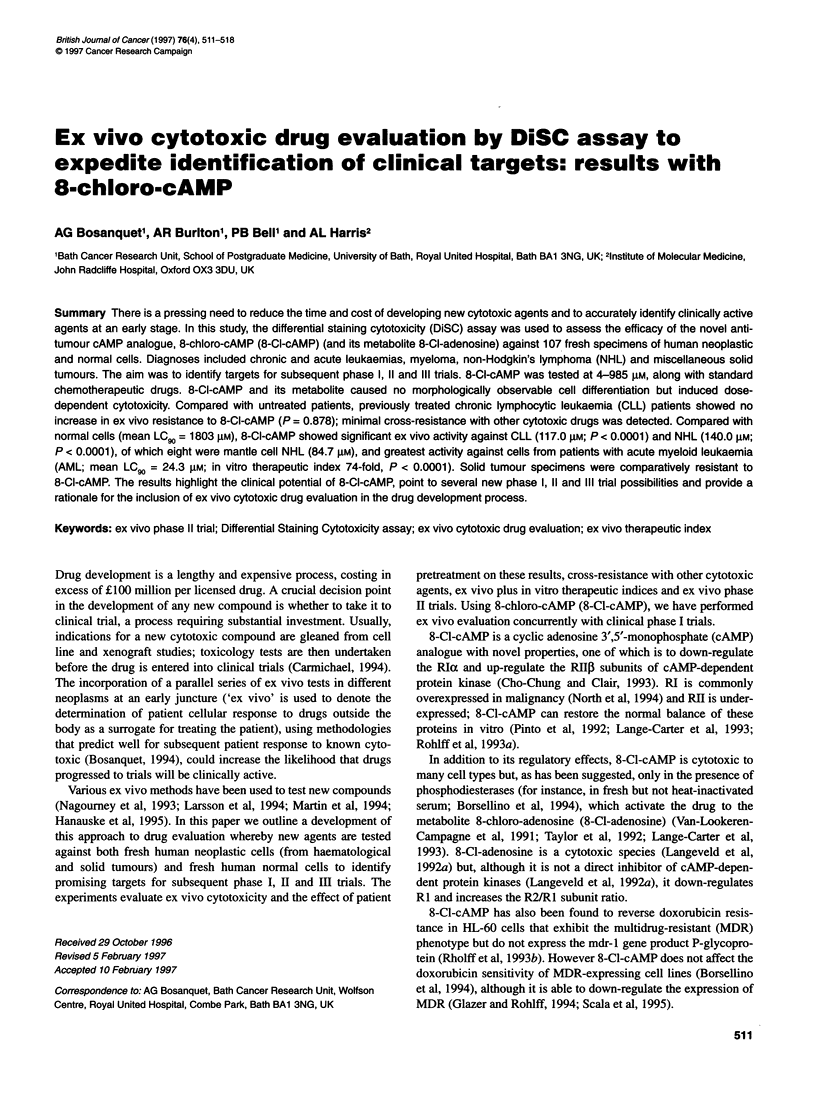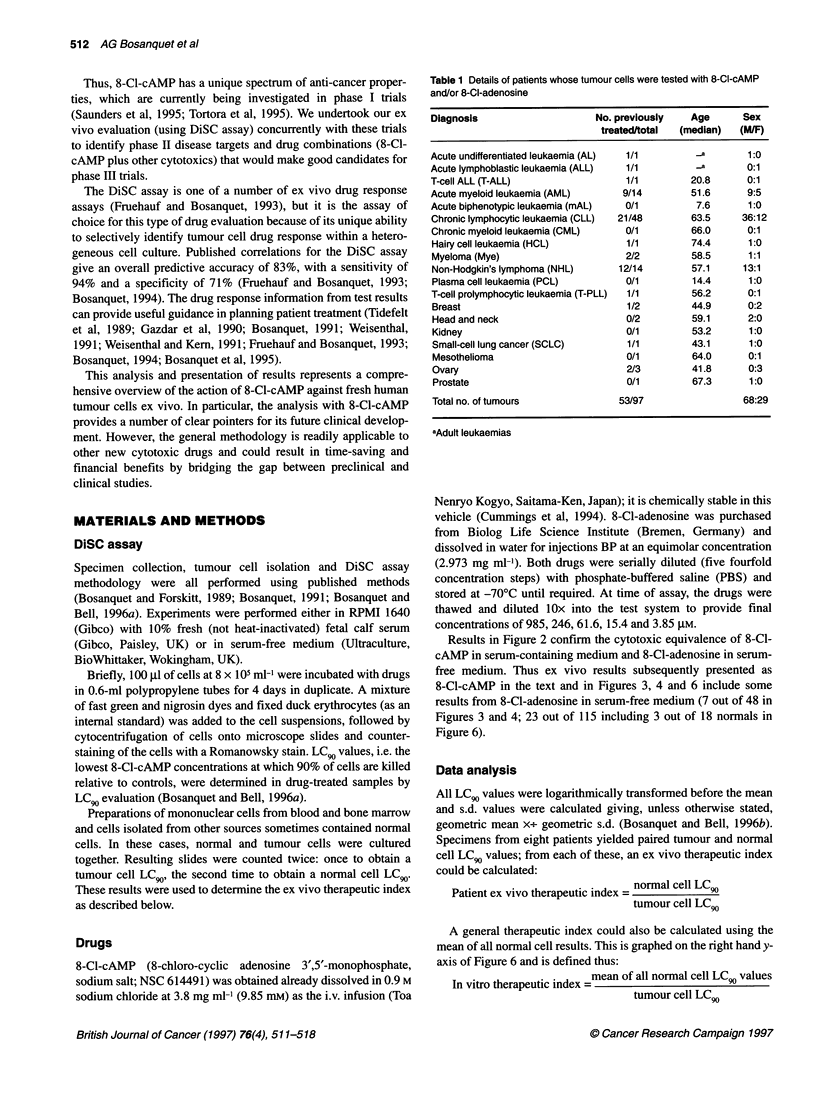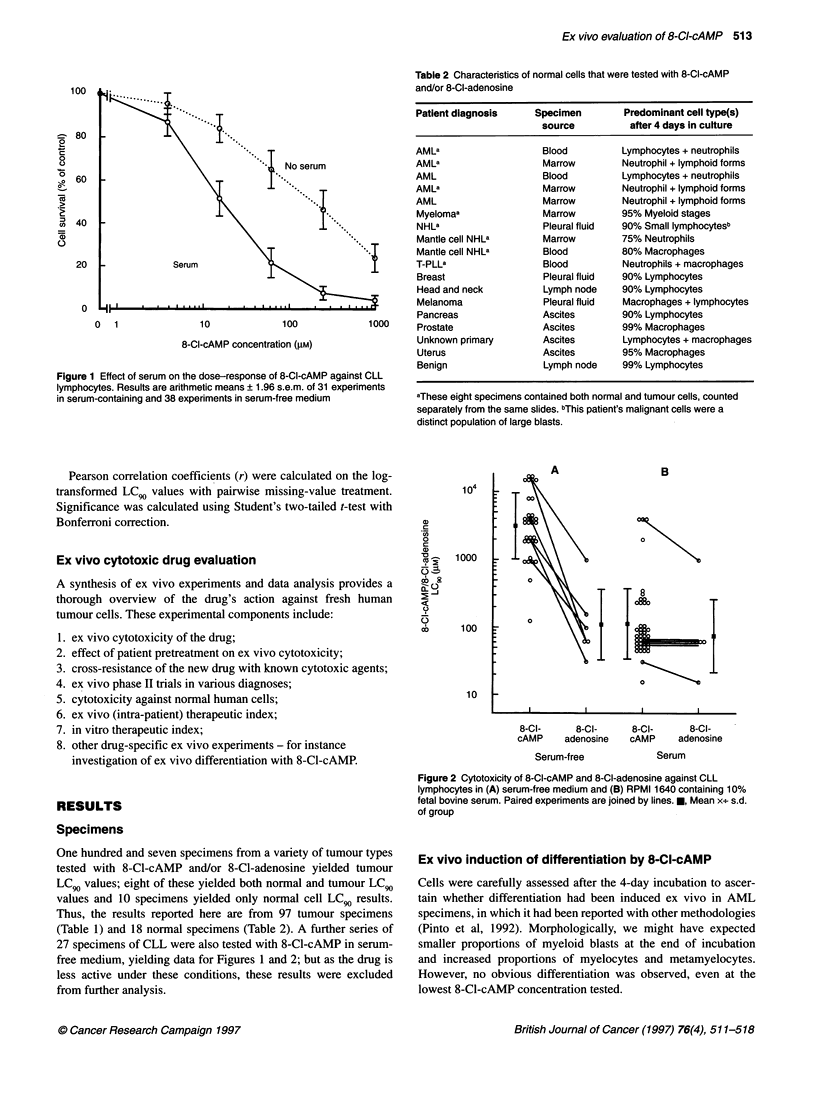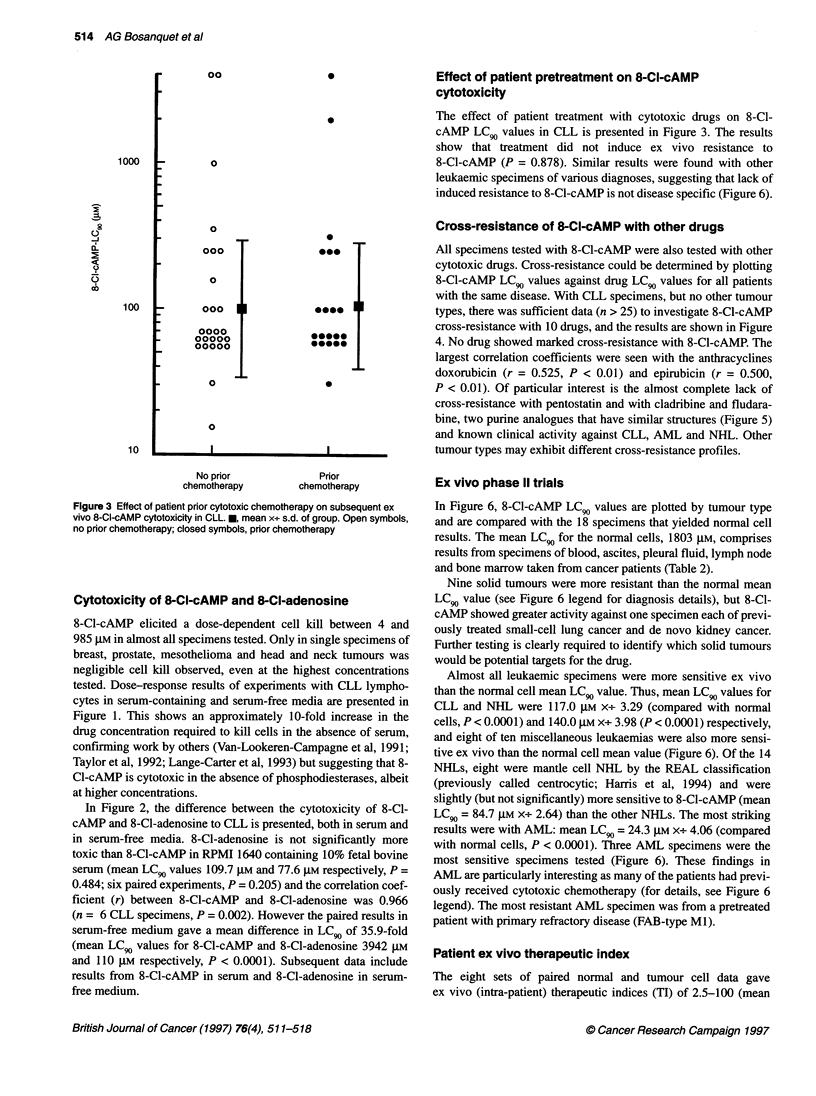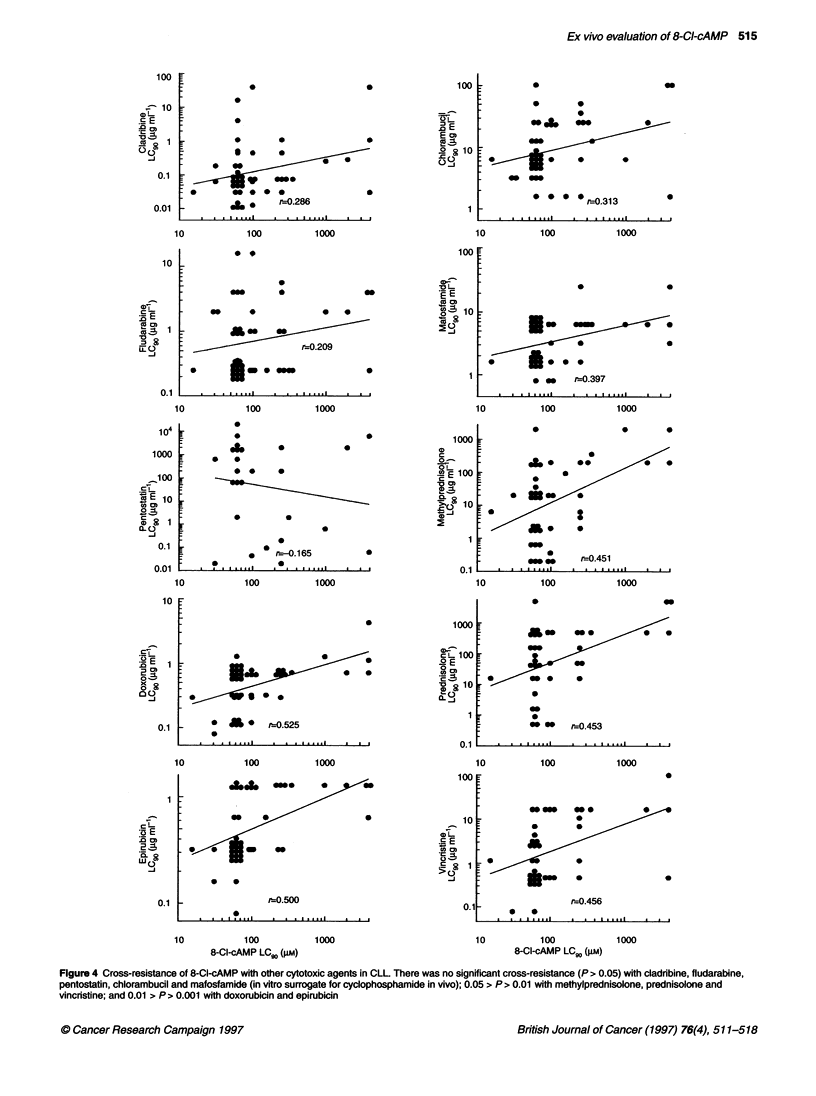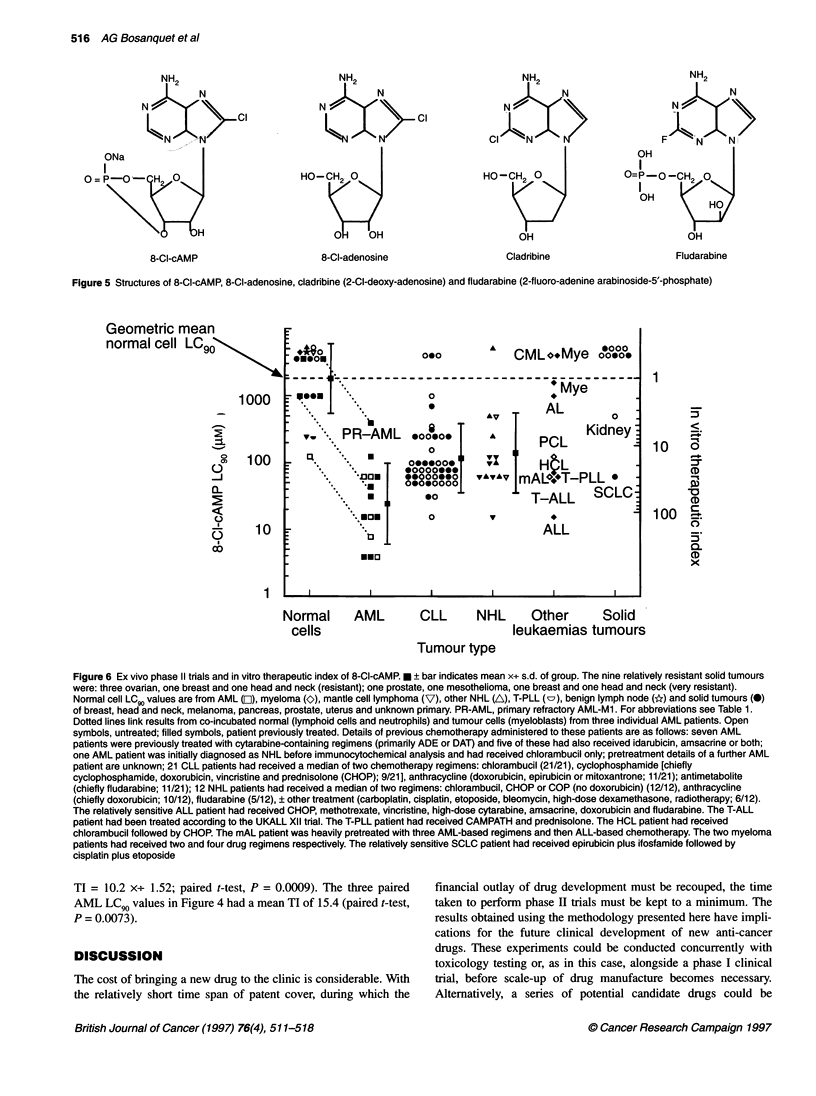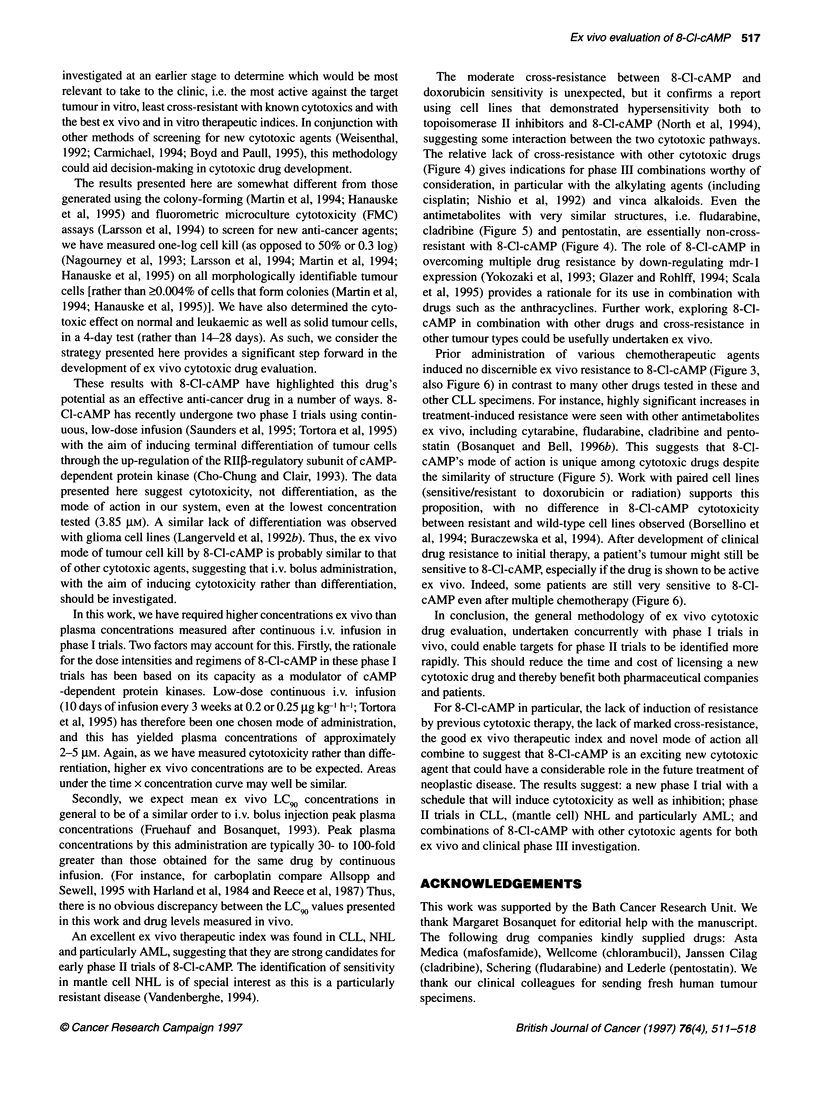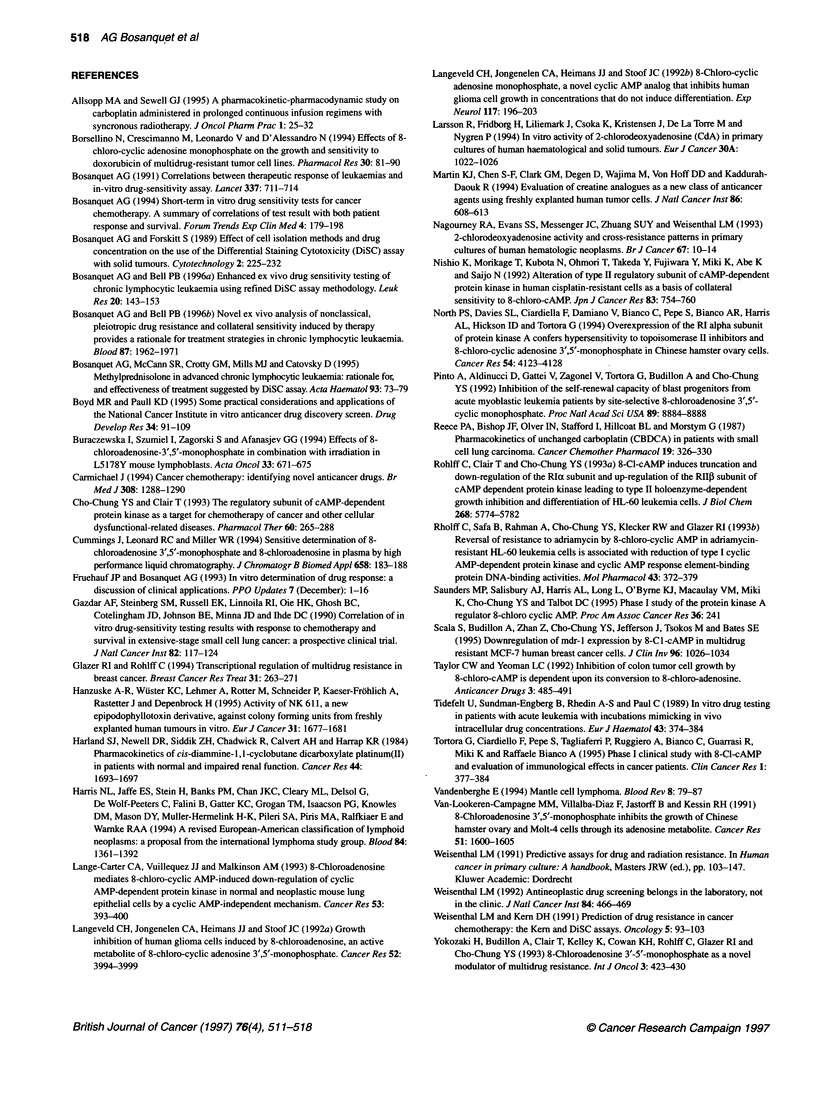# Ex vivo cytotoxic drug evaluation by DiSC assay to expedite identification of clinical targets: results with 8-chloro-cAMP.

**DOI:** 10.1038/bjc.1997.417

**Published:** 1997

**Authors:** A. G. Bosanquet, A. R. Burlton, P. B. Bell, A. L. Harris

**Affiliations:** Bath Cancer Research Unit, School of Postgraduate Medicine, University of Bath, Royal United Hospital, UK.

## Abstract

There is a pressing need to reduce the time and cost of developing new cytotoxic agents and to accurately identify clinically active agents at an early stage. In this study, the differential staining cytotoxicity (DiSC) assay was used to assess the efficacy of the novel antitumour cAMP analogue, 8-chloro-cAMP (8-Cl-cAMP) (and its metabolite 8-Cl-adenosine) against 107 fresh specimens of human neoplastic and normal cells. Diagnoses included chronic and acute leukaemias, myeloma, non-Hodgkin's lymphoma (NHL) and miscellaneous solid tumours. The aim was to identify targets for subsequent phase I, II and III trials. 8-Cl-cAMP was tested at 4-985 microM, along with standard chemotherapeutic drugs. 8-Cl-cAMP and its metabolite caused no morphologically observable cell differentiation but induced dose-dependent cytotoxicity. Compared with untreated patients, previously treated chronic lymphocytic leukaemia (CLL) patients showed no increase in ex vivo resistance to 8-Cl-cAMP (P = 0.878); minimal cross-resistance with other cytotoxic drugs was detected. Compared with normal cells (mean LC90 = 1803 microM), 8-Cl-cAMP showed significant ex vivo activity against CLL (117.0 microM; P < 0.0001) and NHL (140.0 microM; P < 0.0001), of which eight were mantle cell NHL (84.7 microM), and greatest activity against cells from patients with acute myeloid leukaemia (AML; mean LC90 = 24.3 microM; in vitro therapeutic index 74-fold, P < 0.0001). Solid tumour specimens were comparatively resistant to 8-Cl-cAMP. The results highlight the clinical potential of 8-Cl-cAMP, point to several new phase I, II and III trial possibilities and provide a rationale for the inclusion of ex vivo cytotoxic drug evaluation in the drug development process.


British Journal of Cancer (1997) 76(4), 511–518
© 1997 Cancer Research Campaign

### Ex vivo cytotoxic drug evaluation by DiSC assay to expedite identification of clinical targets: results with 8-chloro-cAMP

**AG Bosanquet[1], AR Burlton[1], PB Bell[1] and AL Harris[2]**

[1]Bath Cancer Research Unit, School of Postgraduate Medicine, University of Bath, Royal United Hospital, Bath BA1 3NG, UK; [2]Institute of Molecular Medicine, John Radcliffe Hospital, Oxford OX3 3DU, UK

**Summary** There is a pressing need to reduce the time and cost of developing new cytotoxic agents and to accurately identify clinically active agents at an early stage. In this study, the differential staining cytotoxicity (DiSC) assay was used to assess the efficacy of the novel anti-tumour cAMP analogue, 8-chloro-cAMP (8-Cl-cAMP) (and its metabolite 8-Cl-adenosine) against 107 fresh specimens of human neoplastic and normal cells. Diagnoses included chronic and acute leukaemias, myeloma, non-Hodgkin's lymphoma (NHL) and miscellaneous solid tumours. The aim was to identify targets for subsequent phase I, II and III trials. 8-Cl-cAMP was tested at 4–985 μM, along with standard chemotherapeutic drugs. 8-Cl-cAMP and its metabolite caused no morphologically observable cell differentiation but induced dose-dependent cytotoxicity. Compared with untreated patients, previously treated chronic lymphocytic leukaemia (CLL) patients showed no increase in ex vivo resistance to 8-Cl-cAMP ($P = 0.878$); minimal cross-resistance with other cytotoxic drugs was detected. Compared with normal cells (mean $LC_{90}$ = 1803 μM), 8-Cl-cAMP showed significant ex vivo activity against CLL (117.0 μM; $P < 0.0001$) and NHL (140.0 μM; $P < 0.0001$), of which eight were mantle cell NHL (84.7 μM), and greatest activity against cells from patients with acute myeloid leukaemia (AML; mean $LC_{90}$ = 24.3 μM; in vitro therapeutic index 74-fold, $P < 0.0001$). Solid tumour specimens were comparatively resistant to 8-Cl-cAMP. The results highlight the clinical potential of 8-Cl-cAMP, point to several new phase I, II and III trial possibilities and provide a rationale for the inclusion of ex vivo cytotoxic drug evaluation in the drug development process.

**Keywords:** ex vivo phase II trial; Differential Staining Cytotoxicity assay; ex vivo cytotoxic drug evaluation; ex vivo therapeutic index

Drug development is a lengthy and expensive process, costing in excess of £100 million per licensed drug. A crucial decision point in the development of any new compound is whether to take it to clinical trial, a process requiring substantial investment. Usually, indications for a new cytotoxic compound are gleaned from cell line and xenograft studies; toxicology tests are then undertaken before the drug is entered into clinical trials (Carmichael, 1994). The incorporation of a parallel series of ex vivo tests in different neoplasms at an early juncture ('ex vivo' is used to denote the determination of patient cellular response to drugs outside the body as a surrogate for treating the patient), using methodologies that predict well for subsequent patient response to known cytotoxic (Bosanquet, 1994), could increase the likelihood that drugs progressed to trials will be clinically active.

Various ex vivo methods have been used to test new compounds (Nagourney et al, 1993; Larsson et al, 1994; Martin et al, 1994; Hanauske et al, 1995). In this paper we outline a development of this approach to drug evaluation whereby new agents are tested against both fresh human neoplastic cells (from haematological and solid tumours) and fresh human normal cells to identify promising targets for subsequent phase I, II and III trials. The experiments evaluate ex vivo cytotoxicity and the effect of patient pretreatment on these results, cross-resistance with other cytotoxic agents, ex vivo plus in vitro therapeutic indices and ex vivo phase II trials. Using 8-chloro-cAMP (8-Cl-cAMP), we have performed ex vivo evaluation concurrently with clinical phase I trials.

8-Cl-cAMP is a cyclic adenosine 3′,5′-monophosphate (cAMP) analogue with novel properties, one of which is to down-regulate the RIα and up-regulate the RIIβ subunits of cAMP-dependent protein kinase (Cho-Chung and Clair, 1993). RI is commonly overexpressed in malignancy (North et al, 1994) and RII is under-expressed; 8-Cl-cAMP can restore the normal balance of these proteins in vitro (Pinto et al, 1992; Lange-Carter et al, 1993; Rohlff et al, 1993*a*).

In addition to its regulatory effects, 8-Cl-cAMP is cytotoxic to many cell types but, as has been suggested, only in the presence of phosphodiesterases (for instance, in fresh but not heat-inactivated serum; Borsellino et al, 1994), which activate the drug to the metabolite 8-chloro-adenosine (8-Cl-adenosine) (Van-Lookeren-Campagne et al, 1991; Taylor et al, 1992; Lange-Carter et al, 1993). 8-Cl-adenosine is a cytotoxic species (Langeveld et al, 1992*a*) but, although it is not a direct inhibitor of cAMP-dependent protein kinases (Langeveld et al, 1992*a*), it down-regulates R1 and increases the R2/R1 subunit ratio.

8-Cl-cAMP has also been found to reverse doxorubicin resistance in HL-60 cells that exhibit the multidrug-resistant (MDR) phenotype but do not express the mdr-1 gene product P-glycoprotein (Rholff et al, 1993*b*). However 8-Cl-cAMP does not affect the doxorubicin sensitivity of MDR-expressing cell lines (Borsellino et al, 1994), although it is able to down-regulate the expression of MDR (Glazer and Rohlff, 1994; Scala et al, 1995).

*Received 29 October 1996*
*Revised 5 February 1997*
*Accepted 10 February 1997*

*Correspondence to:* AG Bosanquet, Bath Cancer Research Unit, Wolfson Centre, Royal United Hospital, Combe Park, Bath BA1 3NG, UK

Thus, 8-Cl-cAMP has a unique spectrum of anti-cancer properties, which are currently being investigated in phase I trials (Saunders et al, 1995; Tortora et al, 1995). We undertook our ex vivo evaluation (using DiSC assay) concurrently with these trials to identify phase II disease targets and drug combinations (8-Cl-cAMP plus other cytotoxics) that would make good candidates for phase III trials.

The DiSC assay is one of a number of ex vivo drug response assays (Fruehauf and Bosanquet, 1993), but it is the assay of choice for this type of drug evaluation because of its unique ability to selectively identify tumour cell drug response within a heterogeneous cell culture. Published correlations for the DiSC assay give an overall predictive accuracy of 83%, with a sensitivity of 94% and a specificity of 71% (Fruehauf and Bosanquet, 1993; Bosanquet, 1994). The drug response information from test results can provide useful guidance in planning patient treatment (Tidefelt et al, 1989; Gazdar et al, 1990; Bosanquet, 1991; Weisenthal, 1991; Weisenthal and Kern, 1991; Fruehauf and Bosanquet, 1993; Bosanquet, 1994; Bosanquet et al, 1995).

This analysis and presentation of results represents a comprehensive overview of the action of 8-Cl-cAMP against fresh human tumour cells ex vivo. In particular, the analysis with 8-Cl-cAMP provides a number of clear pointers for its future clinical development. However, the general methodology is readily applicable to other new cytotoxic drugs and could result in time-saving and financial benefits by bridging the gap between preclinical and clinical studies.

**Table 1** Details of patients whose tumour cells were tested with 8-Cl-cAMP and/or 8-Cl-adenosine

| Diagnosis | No. previously treated/total | Age (median) | Sex (M/F) |
|---|---|---|---|
| Acute undifferentiated leukaemia (AL) | 1/1 | –[a] | 1:0 |
| Acute lymphoblastic leukaemia (ALL) | 1/1 | –[a] | 0:1 |
| T-cell ALL (T-ALL) | 1/1 | 20.8 | 0:1 |
| Acute myeloid leukaemia (AML) | 9/14 | 51.6 | 9:5 |
| Acute biphenotypic leukaemia (mAL) | 0/1 | 7.6 | 1:0 |
| Chronic lymphocytic leukaemia (CLL) | 21/48 | 63.5 | 36:12 |
| Chronic myeloid leukaemia (CML) | 0/1 | 66.0 | 0:1 |
| Hairy cell leukaemia (HCL) | 1/1 | 74.4 | 1:0 |
| Myeloma (Mye) | 2/2 | 58.5 | 1:1 |
| Non-Hodgkin's lymphoma (NHL) | 12/14 | 57.1 | 13:1 |
| Plasma cell leukaemia (PCL) | 0/1 | 14.4 | 1:0 |
| T-cell prolymphocytic leukaemia (T-PLL) | 1/1 | 56.2 | 0:1 |
| Breast | 1/2 | 44.9 | 0:2 |
| Head and neck | 0/2 | 59.1 | 2:0 |
| Kidney | 0/1 | 53.2 | 1:0 |
| Small-cell lung cancer (SCLC) | 1/1 | 43.1 | 1:0 |
| Mesothelioma | 0/1 | 64.0 | 0:1 |
| Ovary | 2/3 | 41.8 | 0:3 |
| Prostate | 0/1 | 67.3 | 1:0 |
| Total no. of tumours | 53/97 | | 68:29 |

[a]Adult leukaemias

### MATERIALS AND METHODS

### DiSC assay

Specimen collection, tumour cell isolation and DiSC assay methodology were all performed using published methods (Bosanquet and Forskitt, 1989; Bosanquet, 1991; Bosanquet and Bell, 1996*a*). Experiments were performed either in RPMI 1640 (Gibco) with 10% fresh (not heat-inactivated) fetal calf serum (Gibco, Paisley, UK) or in serum-free medium (Ultraculture, BioWhittaker, Wokingham, UK).

Briefly, 100 μl of cells at $8 \times 10^5$ ml$^{-1}$ were incubated with drugs in 0.6-ml polypropylene tubes for 4 days in duplicate. A mixture of fast green and nigrosin dyes and fixed duck erythrocytes (as an internal standard) was added to the cell suspensions, followed by cytocentrifugation of cells onto microscope slides and counterstaining of the cells with a Romanowsky stain. $LC_{90}$ values, i.e. the lowest 8-Cl-cAMP concentrations at which 90% of cells are killed relative to controls, were determined in drug-treated samples by $LC_{90}$ evaluation (Bosanquet and Bell, 1996*a*).

Preparations of mononuclear cells from blood and bone marrow and cells isolated from other sources sometimes contained normal cells. In these cases, normal and tumour cells were cultured together. Resulting slides were counted twice: once to obtain a tumour cell $LC_{90}$, the second time to obtain a normal cell $LC_{90}$. These results were used to determine the ex vivo therapeutic index as described below.

### Drugs

8-Cl-cAMP (8-chloro-cyclic adenosine 3′,5′-monophosphate, sodium salt; NSC 614491) was obtained already dissolved in 0.9 M sodium chloride at 3.8 mg ml$^{-1}$ (9.85 mM) as the i.v. infusion (Toa Nenryo Kogyo, Saitama-Ken, Japan); it is chemically stable in this vehicle (Cummings et al, 1994). 8-Cl-adenosine was purchased from Biolog Life Science Institute (Bremen, Germany) and dissolved in water for injections BP at an equimolar concentration (2.973 mg ml$^{-1}$). Both drugs were serially diluted (five fourfold concentration steps) with phosphate-buffered saline (PBS) and stored at –70°C until required. At time of assay, the drugs were thawed and diluted 10× into the test system to provide final concentrations of 985, 246, 61.6, 15.4 and 3.85 μM.

Results in Figure 2 confirm the cytotoxic equivalence of 8-Cl-cAMP in serum-containing medium and 8-Cl-adenosine in serum-free medium. Thus ex vivo results subsequently presented as 8-Cl-cAMP in the text and in Figures 3, 4 and 6 include some results from 8-Cl-adenosine in serum-free medium (7 out of 48 in Figures 3 and 4; 23 out of 115 including 3 out of 18 normals in Figure 6).

### Data analysis

All $LC_{90}$ values were logarithmically transformed before the mean and s.d. values were calculated giving, unless otherwise stated, geometric mean ×÷ geometric s.d. (Bosanquet and Bell, 1996*b*). Specimens from eight patients yielded paired tumour and normal cell $LC_{90}$ values; from each of these, an ex vivo therapeutic index could be calculated:

$$\text{Patient ex vivo therapeutic index} = \frac{\text{normal cell } LC_{90}}{\text{tumour cell } LC_{90}}$$

A general therapeutic index could also be calculated using the mean of all normal cell results. This is graphed on the right hand *y*-axis of Figure 6 and is defined thus:

$$\text{In vitro therapeutic index} = \frac{\text{mean of all normal cell } LC_{90} \text{ values}}{\text{tumour cell } LC_{90}}$$

© Cancer Research Campaign 1997

**Figure 1** Effect of serum on the dose–response of 8-Cl-cAMP against CLL lymphocytes. Results are arithmetic means ± 1.96 s.e.m. of 31 experiments in serum-containing and 38 experiments in serum-free medium

**Table 2** Characteristics of normal cells that were tested with 8-Cl-cAMP and/or 8-Cl-adenosine

| Patient diagnosis | Specimen source | Predominant cell type(s) after 4 days in culture |
|---|---|---|
| AML[a] | Blood | Lymphocytes + neutrophils |
| AML[a] | Marrow | Neutrophil + lymphoid forms |
| AML | Blood | Lymphocytes + neutrophils |
| AML[a] | Marrow | Neutrophil + lymphoid forms |
| AML | Marrow | Neutrophil + lymphoid forms |
| Myeloma[a] | Marrow | 95% Myeloid stages |
| NHL[a] | Pleural fluid | 90% Small lymphocytes[b] |
| Mantle cell NHL[a] | Marrow | 75% Neutrophils |
| Mantle cell NHL[a] | Blood | 80% Macrophages |
| T-PLL[a] | Blood | Neutrophils + macrophages |
| Breast | Pleural fluid | 90% Lymphocytes |
| Head and neck | Lymph node | 90% Lymphocytes |
| Melanoma | Pleural fluid | Macrophages + lymphocytes |
| Pancreas | Ascites | 90% Lymphocytes |
| Prostate | Ascites | 99% Macrophages |
| Unknown primary | Ascites | Lymphocytes + macrophages |
| Uterus | Ascites | 95% Macrophages |
| Benign | Lymph node | 99% Lymphocytes |

[a]These eight specimens contained both normal and tumour cells, counted separately from the same slides. [b]This patient's malignant cells were a distinct population of large blasts.

Pearson correlation coefficients ($r$) were calculated on the log-transformed $LC_{90}$ values with pairwise missing-value treatment. Significance was calculated using Student's two-tailed $t$-test with Bonferroni correction.

### Ex vivo cytotoxic drug evaluation

A synthesis of ex vivo experiments and data analysis provides a thorough overview of the drug's action against fresh human tumour cells. These experimental components include:

1. ex vivo cytotoxicity of the drug;
2. effect of patient pretreatment on ex vivo cytotoxicity;
3. cross-resistance of the new drug with known cytotoxic agents;
4. ex vivo phase II trials in various diagnoses;
5. cytotoxicity against normal human cells;
6. ex vivo (intra-patient) therapeutic index;
7. in vitro therapeutic index;
8. other drug-specific ex vivo experiments – for instance investigation of ex vivo differentiation with 8-Cl-cAMP.

### RESULTS

### Specimens

One hundred and seven specimens from a variety of tumour types tested with 8-Cl-cAMP and/or 8-Cl-adenosine yielded tumour $LC_{90}$ values; eight of these yielded both normal and tumour $LC_{90}$ values and 10 specimens yielded only normal cell $LC_{90}$ results. Thus, the results reported here are from 97 tumour specimens (Table 1) and 18 normal specimens (Table 2). A further series of 27 specimens of CLL were also tested with 8-Cl-cAMP in serum-free medium, yielding data for Figures 1 and 2; but as the drug is less active under these conditions, these results were excluded from further analysis.

**Figure 2** Cytotoxicity of 8-Cl-cAMP and 8-Cl-adenosine against CLL lymphocytes in (**A**) serum-free medium and (**B**) RPMI 1640 containing 10% fetal bovine serum. Paired experiments are joined by lines. ■, Mean ×÷ s.d. of group

### Ex vivo induction of differentiation by 8-Cl-cAMP

Cells were carefully assessed after the 4-day incubation to ascertain whether differentiation had been induced ex vivo in AML specimens, in which it had been reported with other methodologies (Pinto et al, 1992). Morphologically, we might have expected smaller proportions of myeloid blasts at the end of incubation and increased proportions of myelocytes and metamyelocytes. However, no obvious differentiation was observed, even at the lowest 8-Cl-cAMP concentration tested.

**Figure 3** Effect of patient prior cytotoxic chemotherapy on subsequent ex vivo 8-Cl-cAMP cytotoxicity in CLL. ■, mean ×÷ s.d. of group. Open symbols, no prior chemotherapy; closed symbols, prior chemotherapy

### Cytotoxicity of 8-Cl-cAMP and 8-Cl-adenosine

8-Cl-cAMP elicited a dose-dependent cell kill between 4 and 985 µM in almost all specimens tested. Only in single specimens of breast, prostate, mesothelioma and head and neck tumours was negligible cell kill observed, even at the highest concentrations tested. Dose–response results of experiments with CLL lymphocytes in serum-containing and serum-free media are presented in Figure 1. This shows an approximately 10-fold increase in the drug concentration required to kill cells in the absence of serum, confirming work by others (Van-Lookeren-Campagne et al, 1991; Taylor et al, 1992; Lange-Carter et al, 1993) but suggesting that 8-Cl-cAMP is cytotoxic in the absence of phosphodiesterases, albeit at higher concentrations.

In Figure 2, the difference between the cytotoxicity of 8-Cl-cAMP and 8-Cl-adenosine to CLL is presented, both in serum and in serum-free media. 8-Cl-adenosine is not significantly more toxic than 8-Cl-cAMP in RPMI 1640 containing 10% fetal bovine serum (mean $LC_{90}$ values 109.7 µM and 77.6 µM respectively, $P$ = 0.484; six paired experiments, $P$ = 0.205) and the correlation coefficient ($r$) between 8-Cl-cAMP and 8-Cl-adenosine was 0.966 ($n$ = 6 CLL specimens, $P$ = 0.002). However the paired results in serum-free medium gave a mean difference in $LC_{90}$ of 35.9-fold (mean $LC_{90}$ values for 8-Cl-cAMP and 8-Cl-adenosine 3942 µM and 110 µM respectively, $P$ < 0.0001). Subsequent data include results from 8-Cl-cAMP in serum and 8-Cl-adenosine in serum-free medium.

### Effect of patient pretreatment on 8-Cl-cAMP cytotoxicity

The effect of patient treatment with cytotoxic drugs on 8-Cl-cAMP $LC_{90}$ values in CLL is presented in Figure 3. The results show that treatment did not induce ex vivo resistance to 8-Cl-cAMP ($P$ = 0.878). Similar results were found with other leukaemic specimens of various diagnoses, suggesting that lack of induced resistance to 8-Cl-cAMP is not disease specific (Figure 6).

### Cross-resistance of 8-Cl-cAMP with other drugs

All specimens tested with 8-Cl-cAMP were also tested with other cytotoxic drugs. Cross-resistance could be determined by plotting 8-Cl-cAMP $LC_{90}$ values against drug $LC_{90}$ values for all patients with the same disease. With CLL specimens, but no other tumour types, there was sufficient data ($n$ > 25) to investigate 8-Cl-cAMP cross-resistance with 10 drugs, and the results are shown in Figure 4. No drug showed marked cross-resistance with 8-Cl-cAMP. The largest correlation coefficients were seen with the anthracyclines doxorubicin ($r$ = 0.525, $P$ < 0.01) and epirubicin ($r$ = 0.500, $P$ < 0.01). Of particular interest is the almost complete lack of cross-resistance with pentostatin and with cladribine and fludarabine, two purine analogues that have similar structures (Figure 5) and known clinical activity against CLL, AML and NHL. Other tumour types may exhibit different cross-resistance profiles.

### Ex vivo phase II trials

In Figure 6, 8-Cl-cAMP $LC_{90}$ values are plotted by tumour type and are compared with the 18 specimens that yielded normal cell results. The mean $LC_{90}$ for the normal cells, 1803 µM, comprises results from specimens of blood, ascites, pleural fluid, lymph node and bone marrow taken from cancer patients (Table 2).

Nine solid tumours were more resistant than the normal mean $LC_{90}$ value (see Figure 6 legend for diagnosis details), but 8-Cl-cAMP showed greater activity against one specimen each of previously treated small-cell lung cancer and de novo kidney cancer. Further testing is clearly required to identify which solid tumours would be potential targets for the drug.

Almost all leukaemic specimens were more sensitive ex vivo than the normal cell mean $LC_{90}$ value. Thus, mean $LC_{90}$ values for CLL and NHL were 117.0 µM ×÷ 3.29 (compared with normal cells, $P$ < 0.0001) and 140.0 µM ×÷ 3.98 ($P$ < 0.0001) respectively, and eight of ten miscellaneous leukaemias were also more sensitive ex vivo than the normal cell mean value (Figure 6). Of the 14 NHLs, eight were mantle cell NHL by the REAL classification (previously called centrocytic; Harris et al, 1994) and were slightly (but not significantly) more sensitive to 8-Cl-cAMP (mean $LC_{90}$ = 84.7 µM ×÷ 2.64) than the other NHLs. The most striking results were with AML: mean $LC_{90}$ = 24.3 µM ×÷ 4.06 (compared with normal cells, $P$ < 0.0001). Three AML specimens were the most sensitive specimens tested (Figure 6). These findings in AML are particularly interesting as many of the patients had previously received cytotoxic chemotherapy (for details, see Figure 6 legend). The most resistant AML specimen was from a pretreated patient with primary refractory disease (FAB-type M1).

### Patient ex vivo therapeutic index

The eight sets of paired normal and tumour cell data gave ex vivo (intra-patient) therapeutic indices (TI) of 2.5–100 (mean

© Cancer Research Campaign 1997

**Figure 4** Cross-resistance of 8-Cl-cAMP with other cytotoxic agents in CLL. There was no significant cross-resistance ($P > 0.05$) with cladribine, fludarabine, pentostatin, chlorambucil and mafosfamide (in vitro surrogate for cyclophosphamide in vivo); $0.05 > P > 0.01$ with methylprednisolone, prednisolone and vincristine; and $0.01 > P > 0.001$ with doxorubicin and epirubicin

8-Cl-cAMP 8-Cl-adenosine Cladribine Fludarabine

**Figure 5** Structures of 8-Cl-cAMP, 8-Cl-adenosine, cladribine (2-Cl-deoxy-adenosine) and fludarabine (2-fluoro-adenine arabinoside-5′-phosphate)

**Figure 6** Ex vivo phase II trials and in vitro therapeutic index of 8-Cl-cAMP. ■ ± bar indicates mean ×÷ s.d. of group. The nine relatively resistant solid tumours were: three ovarian, one breast and one head and neck (resistant); one prostate, one mesothelioma, one breast and one head and neck (very resistant). Normal cell $LC_{90}$ values are from AML (□), myeloma (◇), mantle cell lymphoma (▽), other NHL (△), T-PLL (▿), benign lymph node (☆) and solid tumours (●) of breast, head and neck, melanoma, pancreas, prostate, uterus and unknown primary. PR-AML, primary refractory AML-M1. For abbreviations see Table 1. Dotted lines link results from co-incubated normal (lymphoid cells and neutrophils) and tumour cells (myeloblasts) from three individual AML patients. Open symbols, untreated; filled symbols, patient previously treated. Details of previous chemotherapy administered to these patients are as follows: seven AML patients were previously treated with cytarabine-containing regimens (primarily ADE or DAT) and five of these had also received idarubicin, amsacrine or both; one AML patient was initially diagnosed as NHL before immunocytochemical analysis and had received chlorambucil only; pretreatment details of a further AML patient are unknown; 21 CLL patients had received a median of two chemotherapy regimens: chlorambucil (21/21), cyclophosphamide [chiefly cyclophosphamide, doxorubicin, vincristine and prednisolone (CHOP); 9/21], anthracycline (doxorubicin, epirubicin or mitoxantrone; 11/21); antimetabolite (chiefly fludarabine; 11/21); 12 NHL patients had received a median of two regimens: chlorambucil, CHOP or COP (no doxorubicin) (12/12), anthracycline (chiefly doxorubicin; 10/12), fludarabine (5/12), ± other treatment (carboplatin, cisplatin, etoposide, bleomycin, high-dose dexamethasone, radiotherapy; 6/12). The relatively sensitive ALL patient had received CHOP, methotrexate, vincristine, high-dose cytarabine, amsacrine, doxorubicin and fludarabine. The T-ALL patient had been treated according to the UKALL XII trial. The T-PLL patient had received CAMPATH and prednisolone. The HCL patient had received chlorambucil followed by CHOP. The mAL patient was heavily pretreated with three AML-based regimens and then ALL-based chemotherapy. The two myeloma patients had received two and four drug regimens respectively. The relatively sensitive SCLC patient had received epirubicin plus ifosfamide followed by cisplatin plus etoposide

TI = 10.2 ×÷ 1.52; paired *t*-test, $P = 0.0009$). The three paired AML $LC_{90}$ values in Figure 4 had a mean TI of 15.4 (paired *t*-test, $P = 0.0073$).

### DISCUSSION

The cost of bringing a new drug to the clinic is considerable. With the relatively short time span of patent cover, during which the financial outlay of drug development must be recouped, the time taken to perform phase II trials must be kept to a minimum. The results obtained using the methodology presented here have implications for the future clinical development of new anti-cancer drugs. These experiments could be conducted concurrently with toxicology testing or, as in this case, alongside a phase I clinical trial, before scale-up of drug manufacture becomes necessary. Alternatively, a series of potential candidate drugs could be

© Cancer Research Campaign 1997

investigated at an earlier stage to determine which would be most relevant to take to the clinic, i.e. the most active against the target tumour in vitro, least cross-resistant with known cytotoxics and with the best ex vivo and in vitro therapeutic indices. In conjunction with other methods of screening for new cytotoxic agents (Weisenthal, 1992; Carmichael, 1994; Boyd and Paull, 1995), this methodology could aid decision-making in cytotoxic drug development.

The results presented here are somewhat different from those generated using the colony-forming (Martin et al, 1994; Hanauske et al, 1995) and fluorometric microculture cytotoxicity (FMC) assays (Larsson et al, 1994) to screen for new anti-cancer agents; we have measured one-log cell kill (as opposed to 50% or 0.3 log) (Nagourney et al, 1993; Larsson et al, 1994; Martin et al, 1994; Hanauske et al, 1995) on all morphologically identifiable tumour cells [rather than ≥0.004% of cells that form colonies (Martin et al, 1994; Hanauske et al, 1995)]. We have also determined the cytotoxic effect on normal and leukaemic as well as solid tumour cells, in a 4-day test (rather than 14–28 days). As such, we consider the strategy presented here provides a significant step forward in the development of ex vivo cytotoxic drug evaluation.

These results with 8-Cl-cAMP have highlighted this drug's potential as an effective anti-cancer drug in a number of ways. 8-Cl-cAMP has recently undergone two phase I trials using continuous, low-dose infusion (Saunders et al, 1995; Tortora et al, 1995) with the aim of inducing terminal differentiation of tumour cells through the up-regulation of the RIIβ-regulatory subunit of cAMP-dependent protein kinase (Cho-Chung and Clair, 1993). The data presented here suggest cytotoxicity, not differentiation, as the mode of action in our system, even at the lowest concentration tested (3.85 μM). A similar lack of differentiation was observed with glioma cell lines (Langerveld et al, 1992*b*). Thus, the ex vivo mode of tumour cell kill by 8-Cl-cAMP is probably similar to that of other cytotoxic agents, suggesting that i.v. bolus administration, with the aim of inducing cytotoxicity rather than differentiation, should be investigated.

In this work, we have required higher concentrations ex vivo than plasma concentrations measured after continuous i.v. infusion in phase I trials. Two factors may account for this. Firstly, the rationale for the dose intensities and regimens of 8-Cl-cAMP in these phase I trials has been based on its capacity as a modulator of cAMP -dependent protein kinases. Low-dose continuous i.v. infusion (10 days of infusion every 3 weeks at 0.2 or 0.25 μg $kg^{-1}$ $h^{-1}$; Tortora et al, 1995) has therefore been one chosen mode of administration, and this has yielded plasma concentrations of approximately 2–5 μM. Again, as we have measured cytotoxicity rather than differentiation, higher ex vivo concentrations are to be expected. Areas under the time × concentration curve may well be similar.

Secondly, we expect mean ex vivo $LC_{90}$ concentrations in general to be of a similar order to i.v. bolus injection peak plasma concentrations (Fruehauf and Bosanquet, 1993). Peak plasma concentrations by this administration are typically 30- to 100-fold greater than those obtained for the same drug by continuous infusion. (For instance, for carboplatin compare Allsopp and Sewell, 1995 with Harland et al, 1984 and Reece et al, 1987) Thus, there is no obvious discrepancy between the $LC_{90}$ values presented in this work and drug levels measured in vivo.

An excellent ex vivo therapeutic index was found in CLL, NHL and particularly AML, suggesting that they are strong candidates for early phase II trials of 8-Cl-cAMP. The identification of sensitivity in mantle cell NHL is of special interest as this is a particularly resistant disease (Vandenberghe, 1994).

The moderate cross-resistance between 8-Cl-cAMP and doxorubicin sensitivity is unexpected, but it confirms a report using cell lines that demonstrated hypersensitivity both to topoisomerase II inhibitors and 8-Cl-cAMP (North et al, 1994), suggesting some interaction between the two cytotoxic pathways. The relative lack of cross-resistance with other cytotoxic drugs (Figure 4) gives indications for phase III combinations worthy of consideration, in particular with the alkylating agents (including cisplatin; Nishio et al, 1992) and vinca alkaloids. Even the antimetabolites with very similar structures, i.e. fludarabine, cladribine (Figure 5) and pentostatin, are essentially non-cross-resistant with 8-Cl-cAMP (Figure 4). The role of 8-Cl-cAMP in overcoming multiple drug resistance by down-regulating mdr-1 expression (Yokozaki et al, 1993; Glazer and Rohlff, 1994; Scala et al, 1995) provides a rationale for its use in combination with drugs such as the anthracyclines. Further work, exploring 8-Cl-cAMP in combination with other drugs and cross-resistance in other tumour types could be usefully undertaken ex vivo.

Prior administration of various chemotherapeutic agents induced no discernible ex vivo resistance to 8-Cl-cAMP (Figure 3, also Figure 6) in contrast to many other drugs tested in these and other CLL specimens. For instance, highly significant increases in treatment-induced resistance were seen with other antimetabolites ex vivo, including cytarabine, fludarabine, cladribine and pentostatin (Bosanquet and Bell, 1996*b*). This suggests that 8-Cl-cAMP's mode of action is unique among cytotoxic drugs despite the similarity of structure (Figure 5). Work with paired cell lines (sensitive/resistant to doxorubicin or radiation) supports this proposition, with no difference in 8-Cl-cAMP cytotoxicity between resistant and wild-type cell lines observed (Borsellino et al, 1994; Buraczewska et al, 1994). After development of clinical drug resistance to initial therapy, a patient's tumour might still be sensitive to 8-Cl-cAMP, especially if the drug is shown to be active ex vivo. Indeed, some patients are still very sensitive to 8-Cl-cAMP even after multiple chemotherapy (Figure 6).

In conclusion, the general methodology of ex vivo cytotoxic drug evaluation, undertaken concurrently with phase I trials in vivo, could enable targets for phase II trials to be identified more rapidly. This should reduce the time and cost of licensing a new cytotoxic drug and thereby benefit both pharmaceutical companies and patients.

For 8-Cl-cAMP in particular, the lack of induction of resistance by previous cytotoxic therapy, the lack of marked cross-resistance, the good ex vivo therapeutic index and novel mode of action all combine to suggest that 8-Cl-cAMP is an exciting new cytotoxic agent that could have a considerable role in the future treatment of neoplastic disease. The results suggest: a new phase I trial with a schedule that will induce cytotoxicity as well as inhibition; phase II trials in CLL, (mantle cell) NHL and particularly AML; and combinations of 8-Cl-cAMP with other cytotoxic agents for both ex vivo and clinical phase III investigation.

### ACKNOWLEDGEMENTS

This work was supported by the Bath Cancer Research Unit. We thank Margaret Bosanquet for editorial help with the manuscript. The following drug companies kindly supplied drugs: Asta Medica (mafosfamide), Wellcome (chlorambucil), Janssen Cilag (cladribine), Schering (fludarabine) and Lederle (pentostatin). We thank our clinical colleagues for sending fresh human tumour specimens.

© Cancer Research Campaign 1997

### REFERENCES

Allsopp MA and Sewell GJ (1995) A pharmacokinetic-pharmacodynamic study on carboplatin administered in prolonged continuous infusion regimens with syncronous radiotherapy. *J Oncol Pharm Prac* **1**: 25–32

Borsellino N, Crescimanno M, Leonardo V and D'Alessandro N (1994) Effects of 8-chloro-cyclic adenosine monophosphate on the growth and sensitivity to doxorubicin of multidrug-resistant tumor cell lines. *Pharmacol Res* **30**: 81–90

Bosanquet AG (1991) Correlations between therapeutic response of leukaemias and in-vitro drug-sensitivity assay. *Lancet* **337**: 711–714

Bosanquet AG (1994) Short-term in vitro drug sensitivity tests for cancer chemotherapy. A summary of correlations of test result with both patient response and survival. *Forum Trends Exp Clin Med* **4**: 179–198

Bosanquet AG and Forskitt S (1989) Effect of cell isolation methods and drug concentration on the use of the Differential Staining Cytotoxicity (DiSC) assay with solid tumours. *Cytotechnology* **2**: 225–232

Bosanquet AG and Bell PB (1996*a*) Enhanced ex vivo drug sensitivity testing of chronic lymphocytic leukaemia using refined DiSC assay methodology. *Leuk Res* **20**: 143–153

Bosanquet AG and Bell PB (1996*b*) Novel ex vivo analysis of nonclassical, pleiotropic drug resistance and collateral sensitivity induced by therapy provides a rationale for treatment strategies in chronic lymphocytic leukaemia. *Blood* **87**: 1962–1971

Bosanquet AG, McCann SR, Crotty GM, Mills MJ and Catovsky D (1995) Methylprednisolone in advanced chronic lymphocytic leukaemia: rationale for, and effectiveness of treatment suggested by DiSC assay. *Acta Haematol* **93**: 73–79

Boyd MR and Paull KD (1995) Some practical considerations and applications of the National Cancer Institute in vitro anticancer drug discovery screen. *Drug Develop Res* **34**: 91–109

Buraczewska I, Szumiel I, Zagorski S and Afanasjev GG (1994) Effects of 8-chloroadenosine-3′,5′-monophosphate in combination with irradiation in L5178Y mouse lymphoblasts. *Acta Oncol* **33**: 671–675

Carmichael J (1994) Cancer chemotherapy: identifying novel anticancer drugs. *Br Med J* **308**: 1288–1290

Cho-Chung YS and Clair T (1993) The regulatory subunit of cAMP-dependent protein kinase as a target for chemotherapy of cancer and other cellular dysfunctional-related diseases. *Pharmacol Ther* **60**: 265–288

Cummings J, Leonard RC and Miller WR (1994) Sensitive determination of 8-chloroadenosine 3′,5′-monophosphate and 8-chloroadenosine in plasma by high performance liquid chromatography. *J Chromatogr B Biomed Appl* **658**: 183–188

Fruehauf JP and Bosanquet AG (1993) In vitro determination of drug response: a discussion of clinical applications. *PPO Updates* **7** (December): 1–16

Gazdar AF, Steinberg SM, Russell EK, Linnoila RI, Oie HK, Ghosh BC, Cotelingham JD, Johnson BE, Minna JD and Ihde DC (1990) Correlation of in vitro drug-sensitivity testing results with response to chemotherapy and survival in extensive-stage small cell lung cancer: a prospective clinical trial. *J Natl Cancer Inst* **82**: 117–124

Glazer RI and Rohlff C (1994) Transcriptional regulation of multidrug resistance in breast cancer. *Breast Cancer Res Treat* **31**: 263–271

Hanzuske A-R, Wüster KC, Lehmer A, Rotter M, Schneider P, Kaeser-Fröhlich A, Rastetter J and Depenbrock H (1995) Activity of NK 611, a new epipodophyllotoxin derivative, against colony forming units from freshly explanted human tumours in vitro. *Eur J Cancer* **31**: 1677–1681

Harland SJ, Newell DR, Siddik ZH, Chadwick R, Calvert AH and Harrap KR (1984) Pharmacokinetics of *cis*-diammine-1,1-cyclobutane dicarboxylate platinum(II) in patients with normal and impaired renal function. *Cancer Res* **44**: 1693–1697

Harris NL, Jaffe ES, Stein H, Banks PM, Chan JKC, Cleary ML, Delsol G, De Wolf-Peeters C, Falini B, Gatter KC, Grogan TM, Isaacson PG, Knowles DM, Mason DY, Muller-Hermelink H-K, Pileri SA, Piris MA, Ralfkiaer E and Warnke RAA (1994) A revised European-American classification of lymphoid neoplasms: a proposal from the international lymphoma study group. *Blood* **84**: 1361–1392

Lange-Carter CA, Vuillequez JJ and Malkinson AM (1993) 8-Chloroadenosine mediates 8-chloro-cyclic AMP-induced down-regulation of cyclic AMP-dependent protein kinase in normal and neoplastic mouse lung epithelial cells by a cyclic AMP-independent mechanism. *Cancer Res* **53**: 393–400

Langeveld CH, Jongenelen CA, Heimans JJ and Stoof JC (1992*a*) Growth inhibition of human glioma cells induced by 8-chloroadenosine, an active metabolite of 8-chloro-cyclic adenosine 3′,5′-monophosphate. *Cancer Res* **52**: 3994–3999

Langeveld CH, Jongenelen CA, Heimans JJ and Stoof JC (1992*b*) 8-Chloro-cyclic adenosine monophosphate, a novel cyclic AMP analog that inhibits human glioma cell growth in concentrations that do not induce differentiation. *Exp Neurol* **117**: 196–203

Larsson R, Fridborg H, Liliemark J, Csoka K, Kristensen J, De La Torre M and Nygren P (1994) In vitro activity of 2-chlorodeoxyadenosine (CdA) in primary cultures of human haematological and solid tumours. *Eur J Cancer* **30A**: 1022–1026

Martin KJ, Chen S-F, Clark GM, Degen D, Wajima M, Von Hoff DD and Kaddurah-Daouk R (1994) Evaluation of creatine analogues as a new class of anticancer agents using freshly explanted human tumor cells. *J Natl Cancer Inst* **86**: 608–613

Nagourney RA, Evans SS, Messenger JC, Zhuang SUY and Weisenthal LM (1993) 2-chlorodeoxyadenosine activity and cross-resistance patterns in primary cultures of human hematologic neoplasms. *Br J Cancer* **67**: 10–14

Nishio K, Morikage T, Kubota N, Ohmori T, Takeda Y, Fujiwara Y, Miki K, Abe K and Saijo N (1992) Alteration of type II regulatory subunit of cAMP-dependent protein kinase in human cisplatin-resistant cells as a basis of collateral sensitivity to 8-chloro-cAMP. *Jpn J Cancer Res* **83**: 754–760

North PS, Davies SL, Ciardiella F, Damiano V, Bianco C, Pepe S, Bianco AR, Harris AL, Hickson ID and Tortora G (1994) Overexpression of the RI alpha subunit of protein kinase A confers hypersensitivity to topoisomerase II inhibitors and 8-chloro-cyclic adenosine 3′,5′-monophosphate in Chinese hamster ovary cells. *Cancer Res* **54**: 4123–4128

Pinto A, Aldinucci D, Gattei V, Zagonel V, Tortora G, Budillon A and Cho-Chung YS (1992) Inhibition of the self-renewal capacity of blast progenitors from acute myoblastic leukemia patients by site-selective 8-chloroadenosine 3′,5′-cyclic monophosphate. *Proc Natl Acad Sci USA* **89**: 8884–8888

Reece PA, Bishop JF, Olver IN, Stafford I, Hillcoat BL and Morstym G (1987) Pharmacokinetics of unchanged carboplatin (CBDCA) in patients with small cell lung carcinoma. *Cancer Chemother Pharmacol* **19**: 326–330

Rohlff C, Clair T and Cho-Chung YS (1993*a*) 8-Cl-cAMP induces truncation and down-regulation of the RIα subunit and up-regulation of the RIIβ subunit of cAMP dependent protein kinase leading to type II holoenzyme-dependent growth inhibition and differentiation of HL-60 leukemia cells. *J Biol Chem* **268**: 5774–5782

Rholff C, Safa B, Rahman A, Cho-Chung YS, Klecker RW and Glazer RI (1993*b*) Reversal of resistance to adriamycin by 8-chloro-cyclic AMP in adriamycin-resistant HL-60 leukemia cells is associated with reduction of type I cyclic AMP-dependent protein kinase and cyclic AMP response element-binding protein DNA-binding activities. *Mol Pharmacol* **43**: 372–379

Saunders MP, Salisbury AJ, Harris AL, Long L, O'Byrne KJ, Macaulay VM, Miki K, Cho-Chung YS and Talbot DC (1995) Phase I study of the protein kinase A regulator 8-chloro cyclic AMP. *Proc Am Assoc Cancer Res* **36**: 241

Scala S, Budillon A, Zhan Z, Cho-Chung YS, Jefferson J, Tsokos M and Bates SE (1995) Downregulation of mdr-1 expression by 8-C1-cAMP in multidrug resistant MCF-7 human breast cancer cells. *J Clin Inv* **96**: 1026–1034

Taylor CW and Yeoman LC (1992) Inhibition of colon tumor cell growth by 8-chloro-cAMP is dependent upon its conversion to 8-chloro-adenosine. *Anticancer Drugs* **3**: 485–491

Tidefelt U, Sundman-Engberg B, Rhedin A-S and Paul C (1989) In vitro drug testing in patients with acute leukemia with incubations mimicking in vivo intracellular drug concentrations. *Eur J Haematol* **43**: 374–384

Tortora G, Ciardiello F, Pepe S, Tagliaferri P, Ruggiero A, Bianco C, Guarrasi R, Miki K and Raffaele Bianco A (1995) Phase I clinical study with 8-Cl-cAMP and evaluation of immunological effects in cancer patients. *Clin Cancer Res* **1**: 377–384

Vandenberghe E (1994) Mantle cell lymphoma. *Blood Rev* **8**: 79–87

Van-Lookeren-Campagne MM, Villalba-Diaz F, Jastorff B and Kessin RH (1991) 8-Chloroadenosine 3′,5′-monophosphate inhibits the growth of Chinese hamster ovary and Molt-4 cells through its adenosine metabolite. *Cancer Res* **51**: 1600–1605

Weisenthal LM (1991) Predictive assays for drug and radiation resistance. In *Human cancer in primary culture: A handbook*, Masters JRW (ed.), pp. 103–147. Kluwer Academic: Dordrecht

Weisenthal LM (1992) Antineoplastic drug screening belongs in the laboratory, not in the clinic. *J Natl Cancer Inst* **84**: 466–469

Weisenthal LM and Kern DH (1991) Prediction of drug resistance in cancer chemotherapy: the Kern and DiSC assays. *Oncology* **5**: 93–103

Yokozaki H, Budillon A, Clair T, Kelley K, Cowan KH, Rohlff C, Glazer RI and Cho-Chung YS (1993) 8-Chloroadenosine 3′-5′-monophosphate as a novel modulator of multidrug resistance. *Int J Oncol* **3**: 423–430

© Cancer Research Campaign 1997